# Current Treatment Regimens and Promising Molecular Therapies for Chronic Hepatobiliary Diseases

**DOI:** 10.3390/biom15010121

**Published:** 2025-01-14

**Authors:** Marilena Durazzo, Arianna Ferro, Victor Manuel Navarro-Tableros, Andrea Gaido, Paolo Fornengo, Fiorella Altruda, Renato Romagnoli, Søren K. Moestrup, Pier Luigi Calvo, Sharmila Fagoonee

**Affiliations:** 1 Department of Medical Sciences, University of Turin, C.so A.M. Dogliotti 14, 10126 Turin, Italy; marilena.durazzo@unito.it (M.D.); arianna.ferro@unito.it (A.F.); andrea.gaido@unito.it (A.G.); paolo.fornengo@unito.it (P.F.); 2 2i3T, Società per la Gestione dell’Incubatore di Imprese e per il Trasferimento Tecnologico, University of Turin, 10126 Turin, Italy; victor.navarro@2i3t.it; 3 Department of Molecular Biotechnology and Health Sciences, Molecular Biotechnology Centre “Guido Tarone”, University of Turin, 10126 Turin, Italy; fiorella.altruda@unito.it; 4 General Surgery 2U-Liver Transplant Unit, Department of Surgical Sciences, Azienda Ospedaliero Universitaria Città della Salute e della Scienza di Torino, University of Turin, Corso Bramante 88-90, 10126 Turin, Italy; renato.romagnoli@unito.it; 5 Department of Biomedicine, Aarhus University, 8000 Aarhus, Denmark; sm@biomed.au.dk; 6 Department of Clinical Biochemistry, Aarhus University Hospital, 8000 Aarhus, Denmark; 7 Pediatric Gastroenterology Unit, Regina Margherita Children’s Hospital, Città della Salute e della Scienza, 10126 Turin, Italy; pierluigi.calvo@unito.it; 8 Institute for Biostructure and Bioimaging, National Research Council, Molecular Biotechnology Centre “Guido Tarone”, 10126 Turin, Italy

**Keywords:** liver fibrosis, inflammation, therapeutic interventions, nucleic acid-based therapeutics, stem cells, extracellular vesicles, organoids, mouse models

## Abstract

Chronic hepatobiliary damage progressively leads to fibrosis, which may evolve into cirrhosis and/or hepatocellular carcinoma. The fight against the increasing incidence of liver-related morbidity and mortality is challenged by a lack of clinically validated early-stage biomarkers and the limited availability of effective anti-fibrotic therapies. Current research is focused on uncovering the pathogenetic mechanisms that drive liver fibrosis. Drugs targeting molecular pathways involved in chronic hepatobiliary diseases, such as inflammation, hepatic stellate cell activation and proliferation, and extracellular matrix production, are being developed. Etiology-specific treatments, such as those for hepatitis B and C viruses, are already in clinical use, and efforts to develop new, targeted therapies for other chronic hepatobiliary diseases are ongoing. In this review, we highlight the major molecular changes occurring in patients affected by metabolic dysfunction-associated steatotic liver disease, viral hepatitis (Delta virus), and autoimmune chronic liver diseases (autoimmune hepatitis, primary biliary cholangitis, and primary sclerosing cholangitis). Further, we describe how this knowledge is linked to current molecular therapies as well as ongoing preclinical and clinical research on novel targeting strategies, including nucleic acid-, mesenchymal stromal/stem cell-, and extracellular vesicle-based options. Much clinical development is obviously still missing, but the plethora of promising potential treatment strategies in chronic hepatobiliary diseases holds promise for a future reversal of the current increase in morbidity and mortality in this group of patients.

## 1. Introduction

Chronic hepatobiliary diseases (CHD) are a wide group of illnesses affecting the liver and/or the biliary tract. They are characterized by long-term damage to the tissues, which progressively leads to inflammation, fibrosis, and an increased risk of cancer development (hepatocellular and cholangiocarcinoma). The early injury can be caused by viral infections (hepatitis A virus (HAV), hepatitis B virus (HBV), hepatitis C virus (HCV), hepatitis D virus (HDV), and hepatitis E virus (HEV)), metabolic disorders (metabolic dysfunction-associated steatotic liver disease—MASLD), excessive alcohol intake (alcohol-induced liver disease), or autoimmune response (autoimmune hepatitis—AIH, primary sclerosing cholangitis—PSC, primary biliary cholangitis—PBC) [1].

CHD prevalence is currently estimated to be 1.5 billion and accounts for 4% of deaths worldwide [1,2]. Thus, the prompt identification of the patients and adequate treatment remain a challenge for public health. The plethora of CHD, as well as the factors (genetic, environmental, diet) participating in the development of these diseases, require etiology-specific biomarkers for both CHD diagnosis and prognosis evaluation, as well as the support of genetic testing and advanced molecular imaging strategies to guide personalized CHD management. New potential biomarkers are constantly being discovered with the use of high-throughput molecular biology tools, such as RNA sequencing and proteomics, not only in the form of free-circulating biomolecules but also enclosed in extracellular vesicles (EVs) released upon cellular stress [3,4]. Regarding genetic testing in hepatology, the “when,” “in whom,” and “what” to prescribe require the training of the next-generation hepatologists to interpret genomic analysis data coupled with clinical acumen [5]. For cholangiopathies, for instance, a 52-gene panel that also covers non-coding variants has been implemented to test cholestatic patients with clinical suspicion of Alagille syndrome, citrullinemia type 2, Crigler–Najjar syndrome types 1 and 2, Gilbert syndrome, intrahepatic cholestasis of pregnancy type 3, or progressive familial intrahepatic cholestasis types 1–4, to name but a few (https://blueprintgenetics.com/tests/panels/gastroenterology/cholestasis-panel/, last visited on 1 January 2025). The incorporation of genomic insights into the diagnostic frameworks for assessing liver disease patients with CHD is imperative to provide a correct diagnosis for personalized therapy. Imaging modalities most commonly employed to detect fibrosis and cirrhosis are ultrasound (US), computed tomography, and conventional magnetic resonance imaging (MRI). However, these exhibit low sensitivity in detecting early fibrosis, for which elastography-based US and MRI techniques are more useful [6]. Enhanced sensitivity can be obtained by using probe-assisted molecular imaging. For example, liver inflammation, a precursor for the development of CHD, can be visualized through the oxidatively (reactive oxygen species) activated by the Fe-PyC3A MRI probe [7]. Thus, all these approaches lead to enhanced etiology- and stage-wise diagnosis of CHD, allowing improved disease management.

Despite new findings regarding the pathogenesis of CHDs, early clinical diagnosis and efficient therapies are still scarce. In this review, we discuss both traditional and emerging therapies useful for the treatment of representative metabolic (MASLD), viral (HDV), and autoimmune chronic liver diseases (AIH, PSC, and PBC), with a special focus on emerging molecular targets and therapies with data from animal models and human organoids. We also highlight the potential of nucleic acid-based therapeutics (antisense oligonucleotides, small interference RNAs, and microRNAs mainly) and cell-based therapies (stem cells and extracellular vesicles) for the CHDs considered in this review with the aim of providing an update on this rapidly evolving and very important field. All these aspects, discussed herein, are summarized in Table 1.

### 1.1. Metabolic Dysfunction-Associated Steatotic Liver Disease

The term MASLD has been recently introduced to define a condition characterized by the presence of liver steatosis (≥5%) and at least one of the following conditions: type 2 diabetes mellitus (T2DM), obesity, or metabolic abnormalities (at least two among waist circumference ≥ 102/88 cm, blood pressure ≥ 130/85 mmHg or treated hypertension, triglycerides ≥ 150 mg/dL and HDL-cholesterol < 40/50 mg/dL or lipid medication, prediabetes, insulin resistance, PCR > 2 mg/L) [8].

MASLD is the most common CHD. Its global prevalence ranges from 30.1% to 32.5%, with heterogenic geographic distribution (e.g., higher prevalence in South America, lower in Eastern Europe) due to ethnicity, genetics, and lifestyle factors [1,9,10]. Current epidemiological data are almost doubled if compared to those of the 2000s [9]. However, considering the global spread of obesity and diabetes, the prevalence of MASLD is expected to continue to increase. This fact is not to be undervalued, as MASLD is also associated with a higher risk of mortality. A recent meta-analysis showed an overall mortality of 17.05 per 1000 person-years (95% CI: 10.31–28.05 events) in subjects affected by MASLD, with cardiac-specific mortality of 5.54 per 1000 person-years (95% CI: 2.72–8.35), extrahepatic cancer-specific mortality of 4.21 per 1000 person-years (95% CI: 1.94–6.48), and liver-specific mortality of 1.75 per 1000 person-years (95% CI: 0.58–2.91) [9]. Non-alcoholic steatohepatitis (NASH), recently termed metabolic dysfunction-associated steatohepatitis (MASH), is an advanced and more aggressive form of MASLD, and its global prevalence has been roughly estimated to be around 5% [9]. For simplicity, the terms MASLD and MASH will be adopted hereafter also when considering earlier reports on NAFLD and NASH, respectively.

### 1.2. Hepatitis D Virus (HDV)

HDV is a small defective virus that requires the presence of hepatitis B virus (HBV) to complete its life cycle. In fact, this hepatotropic virus uses HBV-encoded hepatitis B surface antigen (HBsAg) for entry, assembly, and transmission [11].

The global prevalence of HDV infection is around 0.16% in the general population but 4.5% in HBsAg-positive subjects [12]. Considering geographical areas, Mongolia exhibits the highest HDV prevalence (36.9% among HBsAg-positive people), while lower rates are observed in Western regions thanks to the introduction of HBV vaccination and the adoption of other preventive measures [12]. The parenteral route (through blood or blood product exposure) is the main way of HDV transmission. Thus, people at high risk of infection include intravenous drug users, commercial sex workers, promiscuous homosexuals, and HIV-positive patients.

Compared to other viral hepatitis, HDV infection causes the most severe form of disease with rapid progression to cirrhosis and hepatocellular carcinoma (HCC). In fact, HDV-positive patients show an increased risk of decompensated cirrhosis (HR: 3.82, 95% CI: 1.60–9.10), HCC (HR: 2.97, 95% CI: 1.87–4.70), liver transplantation (HR: 7.07, 95% CI: 1.61–30.99), and overall liver-related mortality (HR: 3.78, 95% CI: 2.18–6.56) [13].

### 1.3. Autoimmune Hepatitis (AIH)

AIH is a chronic immune-mediated liver disease. Although the etiopathogenic process is not fully understood, current knowledge suggests that AIH occurs in genetically predisposed subjects after exposure to specific triggering factors (e.g., infections) [14].

AIH is present globally, but its prevalence differs according to sex (higher among women), age (typical bimodal-age-related incidence curve), and ethnicity (lower among Asians, greatest among Caucasians) [15]. AIH is considered a rare disease, as its prevalence ranges from 4.8 to 42.9 per 100,000 people, but rates are continuing to increase [1]. Because of its autoimmune etiology, AIH is frequently associated with other immune-related diseases (such as inflammatory bowel disease—IBD and thyroiditis), and it can also be presented as an overlap syndrome, which has characteristics of both AIH and PBC or AIH and PSC [15,16]. AIH is even associated with a 1.5-fold increased risk of cancer development, both intra- and extra-hepatic (HCC, lymphoma, colorectal), and in case of no response to treatment, a liver transplant is required. Thus, rates of AIH mortality are quite elevated, with 13% of all-cause deaths over 10 years [17]. HCC occurs approximately in 1% of patients and accounts for 27% (7–70%) of liver-related deaths [15,17].

### 1.4. Primary Sclerosing Cholangitis (PSC)

PSC is a chronic disease characterized by progressive inflammation and fibrosis of biliary ducts, which can also lead to cirrhosis and end-stage liver disease development [18]. The etiology of PSC is still unknown, although genetic susceptibility may play an important role. Moreover, some pathogenic theories have been proposed, considering both disease features and prevalence. The peculiar concentric lesions around bile ducts have suggested the presence of defects in mechanisms protecting against bile acid toxicity among PSC patients; the relationship with IBD has also prompted the possible involvement of gut microbiota in disease development [19].

PSC is considered a rare disease. Its prevalence ranges from 0.1 to 31.7 per 100,000, with higher rates reported in North America and Northern Europe [1,15]. Approximately 25% of PSC patients have other autoimmune diseases, and two-thirds of them are affected by IBD [19]. PSC has been associated with an increased risk of overall mortality (RR 3.55, 95% CI: 2.94–4.28), but it particularly raises the risk of cholangiocarcinoma development (RR 584.37, 95% CI: 269.42–1267.51) [20].

### 1.5. Primary Biliary Cholangitis (PBC)

PBC—previously termed “primary biliary cirrhosis”—is a chronic cholestatic autoimmune liver disease characterized by a T-cell lymphocyte-mediated destruction of biliary ducts [21]. Like other biliary diseases, PBC can progressively lead to cirrhosis and liver failure. PBC is considered an autoimmune disease because of the hallmark presence of antimitochondrial antibodies (AMA), which are responsible for the targeted attack of biliary epithelial cells. This response is triggered by a combination of genetic and environmental factors (including cigarette smoking and gut microbiota), causing tolerance breakdown and consequent disease onset [22].

The prevalence of PBC is estimated to be 14.6 per 100,000, with high rates in North America, followed by Europe and Asia [15]. The disease mostly affects women, although recent literature reports that PBC is more prevalent among males than previously thought (F:M 2–4:1 vs. F:M 10:1) [23]. Survival rates for PBC patients under treatment are quite good, amounting to 80% and 77% at 7 and 10 years, respectively [1,24]. Moreover, PBC has been associated with a slightly increased risk of HCC (4.17 per 1000 patient-years), while male sex and advanced liver disease seem to mostly influence its development (15.7 and 9.82 per 1000 person-years, respectively) [25].

## 2. Fibrosis: Pathophysiology and Natural History

Regardless of the *primum movens*, all CHD are united by chronic liver injury, which progressively leads to a fibrotic state. Liver fibrosis comes from the excessive accumulation of extracellular matrix (ECM) in the organ with consequent formation of fibrous scar that can alter both liver parenchyma and function [26].

As fibrosis level correlates with liver dysfunction, it is considered the major risk factor for HCC development and a great determinant of patients’ quality of life. In fact, advanced liver fibrosis (also called cirrhosis) may cause chronic portal hypertension and numerous clinical complications, such as ascites, esophageal varices bleeding, and hepatic encephalopathy [27].

Molecularly, liver fibrogenesis is a complex process. However, despite the etiology, it includes common cellular mechanisms, including hepatocyte death, chronic inflammation, immune response, and hepatic stellate cell (HSC) activation. Understanding these mechanisms is essential in order to develop new anti-fibrotic therapeutic options, which are urgently required for CHDs [28].

**Figure 1:**
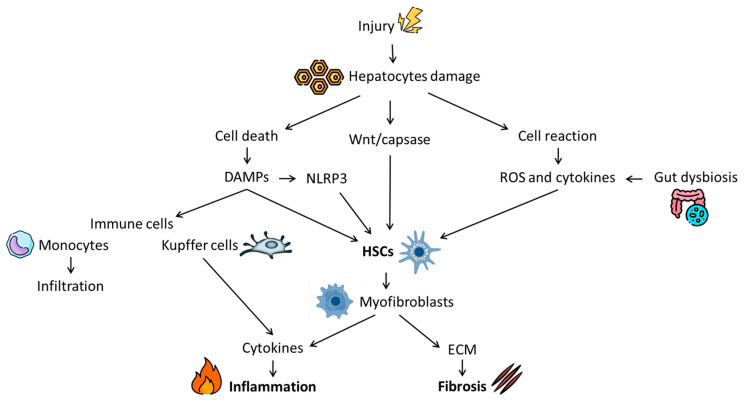
The pathophysiological process of liver fibrosis. DAMPs: danger-associated molecular patterns; NLRP3: NOD-like receptor protein 3 inflammasome; ROS: reactive oxygen species; HSCs: hepatic stellate cells; ECM: extracellular matrix.

Figure 1 schematically shows the pathophysiological process of liver fibrosis.

The toxic, viral, or metabolic factors causing injury to hepatocytes may have either long- or short-term effects. In the case of short-term injury, the pro-fibrogenic process, promoted by damaged cells, is balanced by counteracting anti-fibrotic mechanisms mainly represented by matrix metalloproteinases (MMPs). However, in the case of chronic long-term injury, an imbalance between pro- and anti-fibrogenic mechanisms occurs, causing persistent and excessive ECM production [28]. Specifically, hepatocyte injury can result in two scenarios: cellular death or reaction. In the first case, damaged hepatocytes release intracellular compounds named danger-associated molecular patterns (DAMPs). DAMPs include different molecular groups (e.g., nucleic acids and intracellular proteins) and lead to HSC activation and immune cell recruitment [29]. Alternatively, if the hepatocytes survive, they react by producing inflammatory factors in response to injury. Among these, NADPH oxidase (NOX) is the major source of reactive oxygen species (ROS) and inflammation, which contribute to promoting HSC activation [28].

HSCs play a central role in liver fibrosis. In normal liver, HSCs are quiescent but become activated into a proliferative and contractile phenotype, called myofibroblasts, upon liver damage. During this process, ECM compounds (e.g., collagens and fibronectin) are produced and pro-inflammatory factors are released [30]. The uncontrolled deposition of ECM progressively alters tissue stiffness and flexibility, while pro-inflammatory factors contribute to creating an inflamed environment through the recruitment of immune cells [28,30]. Among cytokines, interleukin 1-beta (IL1-β) and tumor necrosis factor-alpha (TNF-α) act as mere inflammatory agents while transforming growth factor-beta (TGF-β), platelet-derived growth factor (PDGF) and chemokine (C-C motif) ligand 2 (CCL2) also promote HSC division and proliferation [28]. Several inflammatory compounds are also produced by the immune cells, which are retrieved early at the damaged site. Although both innate and adaptive immune systems are involved in the fibrogenic process, the role of lymphocytes is less defined [31]. On the contrary, macrophages play an important part and are considered one of the main ones responsible for hepatic inflammation and perpetuation of the fibrotic state. They include liver resident Kupffer cells and monocyte-derived macrophages [28]. In the early phase of injury, the macrophage population has mostly a pro-inflammatory action. In particular, Kupffer cells start secreting ROS and various cytokines (e.g., IL-1β, TNF-α, PDGF, and CCL2), which stimulate both HSC activation and monocyte infiltration. Moreover, they increase the activity of nuclear factor kappa B (NF-kB) in HSCs, which further promotes pro-inflammatory factor secretion [32]. Activated HSCs, in turn, produce other cytokines that stimulate pro-fibrotic macrophage activity, leading to the amplification and perpetuation of the fibrogenic process [28]. In addition to these mechanisms, two other molecular pathways have been associated with fibrosis: the NOD-like receptor protein 3 inflammasome (NLRP3) and the Wnt/β-catenin signaling. NLRP3 is an intracellular multiprotein complex expressed in Kupffer cells. Stress factors consequent to liver injury (such as DAMPs) can activate NLRP3, promoting HSCs proliferation through the secretion of pro-inflammatory cytokines [33]. Similarly, the Wnt/β-catenin pathway is required for liver development. However, in case of organ damage, Wnt signaling becomes upregulated in HSCs and contributes to fibrogenesis, promoting collagen deposition [34].

In recent years, many studies have also suggested the possible role of gut microbiota in liver fibrosis development due to the close interaction between the gut and liver shaped by the portal vein and the mucosal barrier [35]. The maintenance of the homeostasis of the so-called liver–gut axis is crucial for correct hepatic function, and it is largely related to mucosal barrier integrity. Thus, all factors that alter gut microbiota and/or increase intestinal permeability (e.g., unhealthy diet, alcohol, and toxins) may also promote an inflammatory condition and fibrosis progression [28,35].

In summary, in the case of liver damage, fibrosis is the natural repair response. However, it can also lead to a progressive and uncontrolled deposition of ECM, evolving from a mild (F1) to a more severe stage (F2–F3) of liver fibrosis and, lastly, to cirrhosis (F4) [36]. This condition represents the end stage of any hepatic disease (regardless of origin) and increases the risk of HCC development [26]. Thus, the early detection of the fibrotic state and its prompt treatment are essential to improve patient prognosis. Both traditional and innovative therapies have the common aim of attenuating the fibrogenesis process.

## 3. Traditional Therapies

### 3.1. MASLD

Despite its high prevalence in the global population, there are still few therapeutic options approved for the treatment of CHD. This is especially true for MASLD since, as discussed before, it is a complex medical condition arising from several altered metabolic pathways depending upon environmental, genetic, and behavioral factors [37]. The ideal treatment for MASLD should be able to slow down or even improve liver fibrosis while acting on the underlying metabolic dysfunctions [38]. Currently, clinicians can rely upon different strategies to achieve this result, as follows.

#### 3.1.1. Lifestyle Interventions

It has been proven that healthy nutrition and physical activity synergically protect from the development of MASLD [39]. In a cross-sectional study published in 2022, Vilar-Gomez et al. showed that, among the US population, the MASLD risk was lower in physically active versus inactive patients, with physically active patients defined as ≥600 metabolic equivalent of task [MET] min/week assessed with the Global Physical Activity Questionnaire (OR: 0.71, *p* = 0.043) [40]. Interestingly, Li and colleagues, in a recently published paper, showed that, among the 2017–2020 NHANES cycle participants, only those who participated in leisure-time physical activity (PA) were associated with a reduced risk of MASLD. However, neither occupational PA nor transportation-related PA showed the same protective effects, indicating that increasing overall PA levels should be recommended even for individuals with physically demanding jobs [41]. On the dietary side, several observational studies showed an inverse association of MASLD development with the Mediterranean diet or analogous dietary patterns. Specifically, it appeared that sugar-red meat consumption and sweetened beverages were associated with an elevated risk of developing MASLD in a dose-dependent manner [38].

#### 3.1.2. Pharmacological Therapy

Although recent clinical trials showed promising data (described below), there are few approved MASLD- or MASH-targeted pharmacological therapies. Currently, the best pharmacological treatments clinicians can rely on are weight-lowering drugs such as glucagon-like peptide-1 receptor agonists (GLP1-RAs) since, in case of substantial weight loss, a hepatic histological benefit can be expected [38]. Moreover, the Food and Drug Administration has recently approved the use of *Resmetirom* for the treatment of adults with non-cirrhotic MASLD with moderate to advanced liver fibrosis [42]. Interestingly, this selective thyroid hormone receptor-β agonist showed not only reductions in liver fat content and liver enzyme levels but also improvement in fibrosis risk ratios, with mild side effects (e.g., nausea and diarrhea) [43]. Further studies are needed to investigate both the long-term effects of *Resmetirom* and the development of other pharmacological therapies aimed at the treatment of cardiovascular risk factors, which are very frequent among MASLD patients, such as hypertension, T2DM, and dyslipidemia [38].

#### 3.1.3. Surgical and Endoscopic Therapy

According to the 2024 EASL–EASD–EASO clinical guidelines on the management of MASLD in non-cirrhotic MASLD adults with an approved indication, bariatric surgery should be contemplated as it can boost long-term beneficial effects on the liver. Furthermore, it is associated with remission of T2DM and fewer cardiometabolic events [38]. No other metabolic or bariatric endoscopic procedures are currently advisable as MASLD-targeted therapies.

### 3.2. HDV

The main weapon against HDV hepatitis remains the prevention of infection through HBV vaccination [44]. For those who become infected with HDV, until recently, off-label PEG-IFNα was the only available therapy [45]. Antiviral drugs approved for the treatment of HBV infection showed little to no antiviral effects on HDV since they do not suppress HBsAg production or HDV replication. This has been a major problem for clinicians over the past decades, as only about 50% of HDV+ patients were eligible for PEG-IFNα treatment due to advanced hepatic disease or intolerance. Even eligible patients, however, showed modest results, with undetectable levels of HDV-RNA achieved in less than 50% of treated patients 24 weeks after treatment completion [46]. Those who could not be treated with IFNα could only rely on liver transplants in case of severe hepatic failure [47].

In 2023, Wedemeyer et al. reported the results of a phase 3 randomized trial of a new drug called Bulevirtide for the treatment of chronic HDV infection as a part of the MYR301 study. The trial showed remarkable results, with 56% of the enrolled patients showing liver enzyme normalization and improvement of liver stiffness (−3.08 kPa at 48-week control) [48]. This ground-breaking achievement led to the approval of Bulevirtide by AIFA (Agenzia Italiana del Farmaco), with a therapeutic regimen of 2 mg daily administered subcutaneously.

### 3.3. AIH

All patients with AIH should be treated by pharmacological therapy, with the exception of those with inactive disease documented by clinical, laboratory, and histological assessment. After a large, randomized clinical trial with the corticosteroid budesonide in AIH patients, this drug was found effective and was included as a steroid option in the guidelines on AIH treatment in 2010 [49]. However, in real-life studies, the use of budesonide is limited to AIH patients with low disease activity [50]. Actually, a recent study performed in real-life settings on large cohorts of AIH patients indicated that budesonide treatment resulted in lower remission rates compared to prednisone treatment [50]. The 2019 Practice Guidance and Guidelines from the American Association for the Study of Liver Diseases, published in 2020, recommend a first-line therapy with prednisone or prednisolone 40–60 mg daily for two weeks [51]. After two weeks, when steroid responsiveness is assessed, azathioprine (AZA) can be started (1–2 mg/kg daily), and prednisone can be gradually tapered over the following 6 months with laboratory tests every two weeks to assess responsiveness. Before starting AZA, testing of thiopurine methyltransferase (TPMT) activity is recommended, as patients who have absent TPMT activity (0.3–0.5% of the normal population) are at risk of severe myelosuppression when treated with AZA [52]. Once biochemical remission is achieved, steroids can be discontinued while continuing AZA administration; when prolonged biochemical remission is achieved (>24 months), immunosuppression withdrawal can be considered if the liver biopsy is negative. Higher dosages of steroids are required for the treatment of acute severe AIH, while AZA is not recommended in acute phases [51].

In case of AZA intolerance, the use of mycophenolate mofetil (MMF) as second-line therapy is recommended. However, two recent clinical trials comparing MMF and AZA in AIH patients showed the superiority of MMF in inducing remission, with fewer therapeutic failures [53,54]. Thus, MMF could soon be considered an alternative first choice to AZA, while other drugs such as tacrolimus, rituximab, and infliximab need further evaluation.

### 3.4. PSC

At present, there is no medication approved for the treatment of PSC; several drugs have been studied over the past years, but none of them showed consistent results in altering disease progression [55]. Ursodeoxycholic acid (UDCA) has been widely studied for the treatment of PSC. Its potential beneficial effects include, but are not limited to, increased bile flow, dilution of the bile acid pool, and immunomodulation [56]. Despite these potential benefits, studies using UDCA have shown biochemical improvement (e.g., alkaline phosphatase (ALP) decrease) but no consistent improvement in liver histology or overall survival [57].

Different antibiotics have also been studied in the treatment of PSC, given the potential role of gut dysbiosis in disease progression. Of note, several clinical trials have used oral *vancomycin*, although none of them showed significant improvement in histological or clinical outcomes compared to placebo [57]. Currently, the medical management of PSC focuses on addressing symptoms and complications, such as pruritus. For patients with refractory cholangitis or advanced cirrhosis, liver transplantation remains the only definitive treatment option.

### 3.5. PBC

The recommended first-line treatment for all patients with PBC is oral UDCA at 13–15 mg/kg/day. Its efficacy has been widely studied, and it is considered a very safe medication with minimal side effects (in particular, moderate weight gain and mild gastrointestinal effects) [58]. The combined analysis of three clinical trials involving a total of 548 patients published by Poupon et al. in 1997 showed a one-third reduction in the risk of death or transplant in patients with moderate to severe PBC receiving oral UDCA; interestingly, subgroup analysis did not demonstrate any benefit in patients with stage I/II liver histology [59]. UDCA is usually continued for life.

The only other approved therapeutic option is obeticholic acid (OCA), which directly regulates bile acid metabolism via gene regulation. In a 2015 trial published by Hirschfield et al., OCA as an add-on therapy to UDCA led to a significant decrease in serum ALP from baseline [60]. There are still relatively few data on the long-term efficacy of OCA and its efficacy in different populations, but current evidence has been sufficient for approval in patients with PBC who show inadequate response or intolerance to UDCA [58]. Other drugs, such as budesonide and fibric acid derivatives, are currently under investigation and have not yet been approved in routine clinical practice.

## 4. Emerging Therapeutic Approaches

In recent years, novel anti-fibrotic therapies targeting molecular pathways involved in fibrogenesis have been developed. These molecules can be categorized according to their anti-fibrotic effect and their role in the fibrogenic process. They can act by influencing inflammation pathways, suppressing HSC activation, or regulating ECM deposition [28].

### 4.1. Inflammation Control

The control of inflammation is one of the main anti-fibrotic strategies. Hepatocyte death is a major trigger of inflammation, suggesting that inhibition of liver cell apoptosis may improve both liver inflammation and fibrosis. The pan-caspase inhibitor *Emiric* and the apoptosis signal-regulating kinase (ASK1) inhibitor *Selonsertib* are two anti-apoptotic agents that have been recently tested in liver diseases. In preclinical studies, *Emirican* showed promising results, improving fibrosis and inflammation in MASH murine models [61,62]. However, these effects were not confirmed in clinical trials: a recent meta-analysis stated that *Emricasan* treatment did not provide any benefits in cirrhotic patients [63]. Similarly, the use of *Selonsertib* in animal models led to the improvement of inflammation and suppression of fibrosis progression [64,65]. However, *Selonsertib* therapy had no significant effect on liver biochemistry and progression to cirrhosis in patients with F3 and F4 fibrosis, and two clinical trials were brought to an end early due to lack of efficacy (NCT03053050, NCT03053063) [66].

Another anti-inflammatory strategy is the reduction of oxidative stress, both through ROS scavenging (e.g., vitamin E) or NOX inhibition (e.g., *Setanaxib*). Vitamin E has been widely investigated in MASLD trials. Here, vitamin supplementation showed slight effects on biochemical parameters and liver steatosis, but it was not effective in reducing fibrosis [67,68]. On the contrary, the dual NOX1/4 inhibitor *Setanaxib* improved inflammation markers and fibrosis in both steatotic and cholestatic mice models [69]. This drug also showed anti-cholestatic and anti-fibrotic effects in a preliminary phase 2 trial on PBC patients [70].

As mentioned above, gut microbiota alteration may also promote inflammatory status and fibrosis progression [28]. Thus, several studies have investigated the potential role of probiotics, prebiotics, and symbiotics in liver diseases. In preclinical research, the use of probiotics seemed effective in fibrosis amelioration in both PSC and MASLD models [71,72]. Instead, results from human trials are not so clear. In 2019, a meta-analysis by Liu et al. stated that probiotics and symbiotics supplementation significantly improved biochemical parameters, liver steatosis and stiffness in MASLD patients [32]. However, in more recent clinical trials, treatment with probiotics or symbiotics did not cause significant changes in hepatic fibrosis and only sometimes ameliorated steatosis, but it seemed effective in improving health outcomes among cirrhotic patients [73,74,75]. Conversely, the restoration of gut eubiosis through fecal microbiota transplantation is still under investigation, and so far, only a few small clinical trials have been carried out to evaluate its potential therapeutic role in liver disease [28].

Macrophages are important players both in liver homeostasis as well as in the development of disease, as described above, and can be targeted to dampen inflammation in CHDs. Several nanomedicine formulations have been developed, including regulators of macrophage recruitment or differentiation and polarization, as well as molecular targeting of macrophages, and investigated in mouse models [76,77]. For instance, anti-CD163-directed therapeutic dose delivery of dexamethasone to macrophages in rats fed a high-fructose diet dampened liver damage (including inflammation, fibrosis, and steatosis) [77]. Drugs tilting the balance towards the M2 anti-inflammatory phenotype may block the progression of liver inflammation. The macrophage scavenger function renders the use of drug-loaded nanoparticles in the modulation of macrophage-related pathways an attractive option. Liposomes, lipoplexes, inorganic and organic nanoparticles, and exosomes have been proposed as drug delivery systems for macrophages [78]. For instance, in experimental chronic liver damage induced by carbon tetrachloride (CCl_4_) injection, PEGylated liposomes, without any specific targeting ligand and encapsulating dexamethasone, demonstrated selective accumulation in murine liver macrophages, leading to a decrease in liver injury and fibrosis [79]. Macrophages can also scavenge lipids and dampen inflammation [80]. This function can be harnessed in designing new therapies in the case of MASLD as well as MASH. Macrophage-regulated cell death has an important impact on the progression and resolution of liver diseases (recently described in [81]). Numerous targetable macrophage-related pathways involved in CHD development have been identified and investigated in murine models, but their clinical translation in humans has not always been possible due to species-specific differences in the macrophage system [76].

### 4.2. Hepatic Stellate Cells Inhibition

HSCs play a central role in the fibrogenic process; thus, their inhibition has been considered a promising therapeutic strategy. As mentioned above, the Wnt/beta-catenin pathway is implicated in HSC activation and contributes to liver fibrosis. The inhibition of this signaling pathway has been investigated through two molecules: ICG-001 and PRI-724 [28]. The former acts by disrupting the specific interaction between CREB-binding protein (CBP) and β-catenin, and the outcome in preclinical studies is promising. Akcora et al. observed that ICG-001 significantly inhibited HSC activation and fibrosis progression, both in vitro and in murine models [82]. The use of ICG-001 seemed to mitigate liver fibrosis also in cholestatic mice [83]; however, its effects remain uninvestigated in clinical trials. Similarly, the CB/β-catenin inhibitor PRI-724 showed potential anti-fibrotic properties in murine models of either viral, dysmetabolic, or cholestatic liver diseases [84,85,86]. Recently, an exploratory clinical trial assessing the safety and efficacy of PRI-724 in patients with HCV/HBV-related cirrhosis has been conducted [87]. PRI-724 treatment was well tolerated and caused only mild adverse events (e.g., nausea and diarrhea). However, it has a minor anti-fibrotic effect, with improvement in liver stiffness but not histologically [87].

Another molecule studied for its possible usefulness in anti-fibrotic therapies is the farnesoid X receptor (FXR) [88]. This bile acid-activated nuclear receptor is highly expressed in the liver, especially in HSCs, where it acts as a transcription factor regulating the expression of some pro-fibrotic genes (such as αSMA and TGFβ1) and reducing HSC activation [28,89]. According to recent discoveries, FXR also plays a central role in multiple liver signaling pathways, including bile acid homeostasis, lipid and glucose metabolism, and inflammation [88]. Moreover, FXR activation seems to protect the intestinal mucosal barrier by blocking bacterial translocation and maintaining gut microbiota eubiosis [90]. The prototype for FXR agonists is OCA. Due to its anti-cholestatic properties, OCA is yet to be used as second-line therapy in PBC [91]. However, it has also been recently investigated for its anti-fibrotic effect on different liver diseases. Among MASLD patients, OCA led to a significant improvement in fibrosis histological features; in PBC and PSC subjects, the treatment mainly ameliorated clinical biomarkers (e.g., reduction in ALP levels) [92]. Moreover, several side effects of OCA therapy have been observed, such as pruritus and increased LDL cholesterol [93]. Thus, researchers started investigating other small-molecule FXR agonists without bile acid structural backbones in the hope of avoiding these adverse events. These novel compounds include *Tropifexor* and *Nidufexor*. *Tropifexor* showed significant improvements in ALT, hepatic fat, and fibrosis score (NASH CRN system) among MASLD patients, with pruritus as the most common side effect [94,95]. At the same time, its effect on cholestatic liver diseases has only been observed in animal models [96]. *Nidufexor* is another non-bile acid FXR agonist that showed promising preclinical results [97]. However, information concerning its safety and efficacy in human trials is still not publicly available.

FXR enhancement may also be obtained through another pathway that involves the growth factor fibroblast growth factor (FGF) family. In particular, FGF-19 is synthesized in enterocytes and plays a crucial role in bile acid metabolism, as its production is regulated by the FXR [98]. During the last few years, many FGF19 analogs have been developed to test their possible metabolic effects. Among these, *Aldafermin* is the most studied, and a recent systematic review reported its ability to reduce liver fat content and serum biomarkers (ALT, AST), with diarrhea as the main adverse event. However, *Aldafermin* treatment did not seem effective in improving the histologic features of fibrosis [99]. On the contrary, FGF-21, another member of the FGF family, displayed better anti-fibrotic properties in both animal and human studies [100]. In the systematic review by Jeong et al., the use of different FGF-21 analogs (including *Efruxifermin*, *Pegbelfermin*, and *Pegozafermin*) led to improvements in both steatosis and fibrosis in histological liver samples, with a tolerable safety pattern [101].

### 4.3. Extracellular Matrix Regulation

As mentioned above, the main characteristic of liver fibrosis is the excessive accumulation of ECM. Thus, the regulation of its deposition is another possible target for novel therapies.

Heat shock proteins (HSPs) are defined as stress proteins because their expression is enhanced by environmental and physiological injuries, such as those causing fibrogenesis. In this context, HSPs are crucial for collagen formation and deposition, particularly HSP-47, which acts as a molecular chaperone promoting collagen synthesis and aggregation [102,103]. In preclinical studies, HSP-47 knockdown through small interfering ribonucleic acids (siRNAs) led to a reduction in liver fibrosis in both hepatic and cholestatic models [104]. Similar anti-fibrotic effects were observed in an early clinical trial, where the use of BMS 986263, an HSP47 siRNA-delivering lipid nanoparticle, seemed to ameliorate fibrosis scores in HCV patients [105]. However, studies on collagen inhibitors are further needed to further confirm these preliminary data.

Lysyl oxidases (LOXs) are another group of compounds implicated in ECM and scar development. In the liver, they are prevalently secreted by HSCs and myofibroblasts and promote intramolecular collagen crosslinks through the deamination of lysine residues, contributing to ECM stiffness [106]. Both in vitro and in vivo studies suggest that the inhibition of LOX pathways attenuates hepatic fibrosis and promotes its reversal [107,108]. However, clinical trials testing a LOXL2-blocking antibody (*Simtuzumab*) have not validated preclinical results since no effect was seen on liver fibrosis among patients with MASLD and PSC [109,110].

## 5. Preclinical CHD Models

New preclinical models have become an essential part of the flow of novel therapy testing. In this review, we discuss the most commonly used animal models and organoid systems to reproduce the pathological processes of CHDs. A few of these have been used in the studies considered in this review.

### 5.1. Animal Models

#### 5.1.1. MASLD and MASH

The choice of appropriate animal models is crucial to shed light on the pathophysiological mechanisms underlying MASLD and MASH. The most frequently used rodent models are mice and rats, but the degree of liver steatosis and injury varies. Furthermore, not all human pathological characteristics can be reproduced in rodents. In particular, mice exhibit reduced hepatic steatosis and lower triglyceride levels than observed in humans; conversely, they show more prominent inflammatory damage and increased lipid peroxidation [111]. On the other hand, steatotic stimuli induced by various diets result in more conspicuous steatosis, greater lipid deposition, and more extensive liver injury in males compared to females [112,113]. Diet type, as well as treatment duration, are important variables in the development of features of human MASLD and MASH (discussed extensively in [114] NASH and NAFLD, respectively). Briefly, different high-fat diet formulations exist and usually consist of fat varying from 45% to 71% of total calories (kcal), carbohydrates from 11% to 35%, and protein from 18% to 20% with respect to the normal diet comprising 14% to 18% fat, 58% to 62% carbohydrates, and 22% to 24% protein [114,115,116]. For instance, treatment of C57BL/6J mice with a high-fat diet (Fat: 60%, Carbohydrate: 20%, and Protein: 20%) for 10, 19, 34, and 50 weeks led to an increase in body weight and in insulin and total cholesterol and hepatic triglyceride levels at all time points, with a significant rise in transaminases and inflammatory cell infiltration occurring only after 34 weeks of high-fat diet treatment [115]. Marked steatosis and fibrosis were observed in the liver only after 50 weeks of treatment. Longer treatment for 80 weeks resulted in obesity and insulin resistance, histopathologic features of MASLD, including hepatic steatosis, cell injury, portal and lobular inflammation, fibrosis, hepatic endoplasmic reticulum stress, and gut-bacterial dysbiosis [114,117]. Methionine- and choline-deficient diets fed for 12–16 weeks (sucrose (40% of energy) and fat (10%) lacking methionine and choline) induced steatosis, inflammation, high ALT levels, ballooning of hepatocytes, and MASH-associated features such as ER stress, oxidative stress and autophagocytic stress [118,119]. A high-cholesterol diet (around 1% of the total calorie intake is from cholesterol and often used in combination with high fat (15%) or high cholate (0.5%)) given to C57BL/6J mice for 30 weeks led to obesity, fibrosing steatohepatitis, and hepatic histological and metabolic abnormalities characteristic of MASH [120]. MASH features can also be observed after feeding C57BL/6J mice with a choline-deficient high-fat diet (20% protein, 35% carbohydrate, and 45% fat, without choline added) for 10 weeks, a choline-deficient l-amino acid-defined diet (28.9 kcal/g l-glutamic acid, 15.8 kcal/g l-aspartic acid, 12.7 kcal/g l-arginine, and 10.5 kcal/g l-leucine, without choline bitartrate) in combination with a high-fat diet for 36 weeks, or a high-fat diet and streptozotocin (24.8% protein, 14.4% fat, and 46.7% nitrogen-free extract, supplemented with 200 µg streptozotocin injection) for 7 weeks (of note: HCC develops at 20 weeks) [121,122,123]. On the other hand, a Western diet (21.1% fat, 1.25% cholesterol by weight, 41% sucrose), and a high sugar solution (23.1 g/L d-fructose and 18.9 g/L d-glucose) in conjunction with carbon tetrachloride (injection of 0.32 µg CCl_4_/g body weight) reproduced steatosis, NASH, and fibrosis development in 12 weeks [124]. To closely mimic the human lifestyle slowly leading to MASH, an American Lifestyle-Induced Obesity Syndrome (ALIOS) diet (28% saturated, 57% monounsaturated fatty acids, and 13% polyunsaturated fatty acids), together with high-fructose corn syrup equivalent (55% fructose, 45% glucose by weight) in drinking water at a concentration of 42 g/L) was given to sedentary mice. These mice developed steatosis after 26 weeks of treatment and a NASH-like phenotype (increased body weight and fat mass, hyperlipidemia, and insulin resistance) after 52 weeks of treatment [125]. The current dietary animal models can reproduce only some features of the human MALFD, which in some cases can progress to MASH, thus allowing the study of the physiopathology and progression of MASLD in a stage-wise fashion. The choice of mouse strains is also an important determinant in accurately reproducing MASLD and MASH, with recent studies showing that the FVB/N mouse strain is the most appropriate diet-induced mouse model for the recapitulation of these CHDs [126].

There are also genetic mouse models, such as *ob*/*ob* mice (autosomal recessive mutation in the leptin gene) and *db*/*db* mice (autosomal recessive diabetic gene (*db*), bearing mutations in the *leptin* gene) [127]. Both *ob*/*ob* and *db*/*db* mice are remarkably obese, and they manifest severe insulin resistance, hyperinsulinemia, and hyperglycemia as metabolic features. Nevertheless, due to the unstable expression of leptin levels in *db*/*db* mice, they develop more severe diabetes than *ob*/*ob* mice but have less hepatic steatosis than the latter [127]. In genetically modified rat models, such as the leptin-receptor-deficient obese Zucker *fa*/*fa* rats, obesity, hyperlipidemia, and impaired insulin metabolism develop compared to the lean Zucker rats [128]. The former can be used for evaluating approaches to counteract MASLD [129].

Non-human primates, like rhesus macaques and crab-eating macaques, are frequently utilized in liver disease studies because of their genetic (sequence homology is up to 93%) and physiological resemblances to humans [130,131,132,133,134,135]. These primates offer the benefit of naturally developing obesity, exhibiting physiological and biochemical traits that closely mirror those seen in humans, such as body mass index, fat buildup in the abdomen, insulin resistance, altered lipid profiles, and the onset of diabetes mellitus. However, the use of non-human primates in scientific research is limited by ethical and regulatory issues worldwide.

#### 5.1.2. HDV

Besides humans, HDV can naturally co-infect chimpanzees [136]. Experiments in these animals have uncovered crucial insights into the initiation of HDV infection and viral biology. In particular, chimpanzees with chronic HBV and superinfected with HDV develop acute, self-limited type D hepatitis and with susceptibility to reappearance of HDV replication [136,137]. Moreover, these models demonstrated distinct cytokine profiles associated with disease severity and clinical outcome [138]. However, due to ethical concerns, the National Institutes of Health (NIH) avoids the use of chimpanzees in research. Successively, researchers have made efforts to identify alternative animal models. Some studies have demonstrated that other non-human primate models, such as monkey baboons, monkey tamarins, and crab-eating monkeys (*Macaca fascicularis*), do not support HDV binding and infection due to differences in their sodium taurocholate co-transporting polypeptide (NTCP) protein sequence [139,140]. New strategies based on the transduction of primary hepatocytes obtained from different species (mouse, rat, dog, pig, and macaque) with adeno-associated viral vectors encoding human NTCP in macaques made the establishment of HDV infection possible in vitro [141]. However, the limited availability of hepatocytes, especially from non-human primates, genetic diversity, high costs, and challenges in conducting experiments limit their applicability for future HDV infection research.

An alternative to non-human primates is represented by murine models (mice and rats). Transgenic mice expressing partial or complete viral genomes are the most common model to study viral replication. However, the viral life cycles are incomplete, and immunopathology cannot be investigated. To overcome these limitations, novel humanized mouse models bearing human hepatocytes, with and without humanized immune systems, have been developed as hosts for hepatotropic viruses [142,143,144,145]. In particular, the use of uPA/SCID mice harboring chimeric humanized livers has demonstrated that HBV/HDV co-infections and superinfections can be established in vivo [146]. This model mimics the pathophysiology in humans, where co-infection induces innate antiviral responses such as HLA class I molecules, ISGs, pathogen recognition sensor TLR2, DNA cytosine deaminases, and TGF-β and is useful for drugs [147,148]. Nevertheless, it is important to note that immune-competent animal models are necessary for a deeper elucidation of mechanisms regulating cellular mediators of the antiviral response. This is important because, during viral infections, the immune response is critical for pathogen persistence, its clearance, and the progression of disease-associated pathologies. Humanized mice models might be the solution in this field [149,150]. In humans, excessive tissue scarring activates HSCs, contributing to fibrotic processes, which take decades to develop and progress in humans. The humanized mice models have the advantage of presenting a faster evolution of the pathology, with accelerated development of fibrosis, hence offering the opportunity to further investigate its etiology and ways to counteract it [151,152,153]. Moreover, humanized mice represent valuable models for studying robust hepatitis virus infections, enabling a closer approximation of the virological parameters observed in patients [152,153].

#### 5.1.3. AIH

The AIH animal models represent a crucial strategy for drug screening, as they can provide essential information about the mechanisms of AIH and the basis for further preclinical trials. There are two different classes of AIH animal models: spontaneous (naturally occurring or knockout) and induced (by biological or physicochemical factors). The AIH models provide information about hepatic immunology due to their similarity with human disease, hence aiding in the evaluation of novel therapeutic strategies. Nevertheless, autoimmune disorders in mice have different autoantigen targets with respect to humans, and inducing comparable chronic recurrent AIH is a substantial challenge in these animals [154]. These models aim to enhance understanding of immune system interactions in the liver, as well as to pinpoint the key factors that drive the progression of the disease.

Most of the presently employed humanized models for autoimmune diseases make use of immunodeficient mice that are repopulated with human immune cells. Examples of immunodeficient mice include the NOD.Cg-PrkdcscidIl2rgtm1Wjl (NSG) mouse, NOD.cg-PrkdcscidIl2rgtm1Sug (NOG) mouse, NOD.Cg-Rag1tm1MomIl2rgtm1Wjl (NRG) and the BRG mice (BALB/c/Rag1−/− IL-2rγ−/− and BALB/c/Rag2−/− IL-2rγ−/−) [155,156]. These mice offer the great advantage of allowing almost all lineages of human immune cells to be investigated. The humanized models using repopulated immunodeficient mice expressing HLA class I or II bring the models even closer to the human situation but are complex and require experimental effort [155].

Several mechanisms have been proposed for the development of AIH, including genetic predisposition, environmental triggers, as well as the involvement of liver sinusoidal endothelial cells (LSEC) in the initial T cell-mediated sinusoidal endothelium injury and defects in regulatory T cells (Treg) functions [157,158]. Lately, although extremely rare, post-COVID-19 vaccination AIH was reported in a few cases [159,160,161]. To further investigate AIH pathology, mice models have been developed. The most commonly used AIH animal models are generated using S100 (supernatant from syngeneic liver homogenate centrifuged at 100,000× *g* and emulsified in complete Freund’s adjuvant) and concanavalin A [162,163]. The S100-based model is simpler to generate and reproduces certain features that are characteristics of human AIH in C57BL/6 mice, such as perivascular cellular infiltrates, hepatocyte necrosis, and S-100 protein-specific T-cell formation [162,164]. Moreover, genetically modified mice overexpressing specific proteins, such as those showing liver-specific expression of the MHC class I Kb antigen, were employed to study how self-tolerance can be disrupted [165]. Some studies have revealed, using knock-out mice, new findings associated with AIH immunopathology. For instance, the loss of some components, such as Tregs and PD-1-mediated signaling, leads to lethal AIH, while the increased expression of PD-L1 and B7-DC on Kupffer cells and LSECs can lead to the binding of T cells through PD-1, thus suppressing autoreactive lymphocytes, which regulate the progression of AIH [166,167]. Furthermore, these models have evidenced that loss of PD-1 resulted in enhanced proliferative capacity of T cells in the viral liver [168]. Additionally, mice with mutations in TGF-β display multi-organ inflammatory damage, where the liver is the most susceptible to spontaneous and extensive damage; hepatocyte necrosis with a mechanism involving the IFN γ-mediated immunity occurs, which is similar to humans [169,170,171].

#### 5.1.4. Mice Models of PBC and PSC

Various murine models of PBC have been developed, providing valuable tools for understanding disease mechanisms, identifying new therapeutic targets, and advancing drug discovery. As with AIH, these models are generally classified into two categories: spontaneous and induced. While both types share similarities with human PBC, they also exhibit distinct features. Spontaneous models include Non-Obese Diabetic (NOD) mice, dominant-negative TGF-β receptor II (dnTGFβRII) mice, IL-2 receptor α knockout (IL-2Rα−/−) mice, FoxP3-deficient mice, AE2 gene-disrupted (AE2a,b−/−) mice, and ARE-Del−/− mice [172]. Conversely, induced models are especially valuable for investigating specific pathogenic factors in PBC. Examples include mice immunized with 2-Octynoic acid-BSA (2-OA-BSA), Poly I:C-sensitized mice, and those exposed to *Novosphingobium aromaticivorans*. Both spontaneous and induced models serve as indispensable resources for deepening our understanding of PBC and facilitating the development of novel therapeutic approaches [172,173].

The experimental models of PSC do not fully reproduce the complex pathobiology and natural history of PSC, which include the surge of fibrous scarring and obliteration in both intrahepatic and extrahepatic bile ducts linked to intestinal inflammation, degeneration of biliary epithelial cells, increased likelihood of cholangiocarcinoma development, and preferred male-biased replication [174]. The currently employed models of PSC comprise the (1) chemically induced cholangitis caused by the administration of 2,4,6-trinitrobenzene sulfonic acid (TNBS) or by 3,5-diethoxycarbonyl-1,4-dihydrocollidine (DDC) feeding; (2) infection with bacteria or bacterial products (e.g., infection of the biliary tract with *Cryptosporidium parvum* or of the gastrointestinal tract with *Helicobacter* species, such as *H. hepaticus*) inducing severe cholangitis and biliary fibrosis in immunodepressed mice, and cholangitis and ductular reaction, respectively; (3) transgenic mouse models (e.g., the *Mdr2*^−/−^ mice with the targeted disruption of the *Abcb4* gene involved in phosphatidylcholine transport from the hepatocytes into bile) [174,175,176,177,178]. Most recently, cholangiocyte-specific genetic deletion of kindlin-2 (a tight junction stabilizer acting between cholangiocytes) in mice led to the typical onion skin-type fibrosis, bringing the model a step closer to human PSC for future studies [179].

### 5.2. Organoids for Liver Diseases Modelling

In recent decades, preclinical trials have relied on experimental animals, stem cells, and two-dimensional (2D) cell culture systems as platforms for evaluating new drug candidates. However, in the last decade, these approaches gave way to the 3D in vitro cell culture models, organoids, and organ-on-a-chip models [180]. Organoids are organ-specific cell cultures generated from pluripotent stem cells (PSCs) and/or multipotent adult stem cells (ASCs) and/or differentiated primary cells to mimic the architecture and function of native tissues [181,182,183,184]. The cells forming organoids are able to self-organize and differentiate into functional organ-specific cell types, exhibiting highly physiological properties resembling the cell differentiation capacity as well as cell–cell and cell–matrix interactions [185,186].

Human organoids representing several human organs, such as the intestine, stomach, and liver, in vitro (both hepatocyte and biliary organoids) have been developed [181,182,187,188]. These hepatobiliary organoids are currently being used to study key processes during liver development, to model diseases, and to identify the core pathological mechanisms [189].

Organotypic liver models are known for their strong liver functionality, realistic expression of drug-metabolizing enzymes, and drug-transport proteins, making them valuable tools for reliably predicting hepatotoxicity [190]. Hepatocyte organoids express both the phase I drug-metabolizing cytochrome P450 (CYP) and the phase II detoxification enzymes at comparable levels to liver tissue transcriptomic profiles [191,192]. These and other characteristics make organoids an important tool for toxicological outcome prediction, and they, therefore, represent a valuable tool for toxicology and pharmacology tests [193]. To investigate the mechanisms involved in fibrosis progression in the liver, 2D cell cultures have been mostly used [194]. Nevertheless, as mentioned above, 2D cultures are not the best approach to elucidate the pathophysiological mechanism in disease progression. Organoid technology offers, again, an advantage in fibrosis in vitro modeling, as it allows for the co-cultivating of hepatocytes and non-parenchymal cells involved in fibrosis progression [195]. Hepatocytes and cholangiocytes grown into organoid systems also show polarization, a process that is essential for proper liver function. The hepatocytes are in contact with blood on the basolateral side (for uptake of xenobiotics, for example) and form the bile canaliculus on the apical side (for excretion of the metabolized endo- and xenobiotics into bile) [196]. Cholangiocytes, too, are polarized cells with well-organized apical and basal membranes that ensure the formation of primary cilia, P-glycoprotein-mediated transport, and farnesoid X receptor (FXR)-dependent functions [197].

A more sophisticated method combines both liver organoids and organ-on-a-chip technologies (liver-on-chip). The liver-on-a-chip technology further merges microchip technology with a microfluidic perfusion system that allows it to mimic the liver structure and function in a more resembling situation [198]. In particular, this technology has been used to study, among others, the non-alcoholic fatty liver disease [199].

#### 5.2.1. MASLD

The heterogeneity and complexity of MASLD and MASH, as well as the multiple pathways and factors involved in disease progression, have limited the development of curative drugs to treat these conditions. Most of the previous studies were based on animal models [200,201]. However, due to significant species differences in architecture, regenerative mechanisms, disease progression, inflammatory markers, metabolism rates, and drug responses, these models have failed to fully replicate the exact etiology, pathogenesis, and severity of MASH progression in humans [202,203,204].

Considering the diverse cell types involved in the development of MASLD and MASH, the ideal liver organoid model for studying these diseases would be constructed using multilineage systems. Utilizing iPSCs from various donors, which include cells from different lineages like hepatocytes, HSCs, BECs, and KCs, enables the creation of organoids that display steatosis, inflammation, and fibrosis when exposed to free fatty acids. This approach is highly valuable for creating multicellular organoids that replicate the pathogenesis of MASH [205].

For MASH and MASLD, organoid technology has contributed to a revolution in disease modeling. In this approach, hepatic organoids are typically generated using hepatocyte cell lines like HepG2 or primary human hepatocytes, either alone or in combination with biliary epithelial cells (BECs) and nonparenchymal cells such as KCs, HSCs, and LSECs. These organoids, cultured with fatty acids, have been utilized to explore the molecular processes driving fibrosis and to evaluate drug candidates targeting specific pathological pathways of MASH and MASLD [206,207]. Organoids generated from other cell sources, such as iPSCs, have been used with great success in exploring the pathology of liver diseases and facilitating the investigation of biological processes governing biliary development, disease modeling, and drug testing [208].

#### 5.2.2. PBC and PSC

Cholangiocyte organoids (COs) exhibit both ductal and cyst-like structures, maintaining apical–basal polarity. These organoids are biologically active and serve as a crucial model for studying the molecular mechanisms behind biliary diseases [182,184,209,210]. The application of COs in the study of cholangiopathies and bile-related disorders shows significant potential. They can be utilized in basic research to study liver cell differentiation but also in the modeling of cholangiopathies by recreating the physiological microenvironment [211,212]. COs offer promising models for investigating the pathophysiology of PSC and could serve as platforms to evaluate compounds aimed at managing the inflammatory phenotype. This is supported by the fact that COs preserve the donor’s genetic and morphologic characteristics [188,213]. They share molecular properties, growth rate, expression of biliary markers, functional properties, and upregulation of genes related to immune regulation in PSC-derived organoids, comparable to cholangiocytes of PSC tissue [189]. Hepatobiliary organoids are useful for screening drug sensitivity/resistance for cholangiocarcinoma in vitro and show similar performance and efficacy in the patients from whom the organoids were derived (IHCC) [214]. Hepatobiliary organoid models can also be employed to study the HDV life cycle, replicate critical aspects of the disease, and identify potential treatments through drug screening.

## 6. Promising Targeted Therapies in CHD

Interconnected and complex molecular pathways, not only in the liver but also in distant organs, are responsible for maintaining a balanced hepatobiliary homeostasis and function. Dysregulated expression of any component can lead to aberrant signaling and pathogenetic changes. Thus, potentially, any specific molecule or molecular pathway can become a therapeutic target in chronic liver diseases. Targeted therapy has the advantage of acting specifically against disease-causing cells or processes, hence reducing side effects to a minimum [215]. Due to poor pharmacokinetics or toxicity concerns, various therapies undergoing clinical trials for the treatment of hepatic inflammation have failed. Current principal modalities include nucleic acid-based therapeutics, small molecule inhibitors, stem cell therapy, extracellular vesicles (EVs), and nanotechnology, offering disease-specific precision. Most drugs have a natural tropism for the liver. In fact, the liver is the most favorite off-target organ destination of injected compounds or cells. We discuss herein the current progress made in nucleic-acid-based research in the context of CHD.

### 6.1. Nucleic Acid-Based Therapeutics

Nucleic acid-based therapies harness genetic material technologies to modulate disease-associated genes and pathways. In the last decade, several DNA- or RNA-based therapies have been issued for chronic human diseases, including the liver [216]. These modern therapies include mRNA therapy, gene silencing, genome and epigenome editing, and gene restoration. Gene silencing technologies include RNA interference (RNAi), in which small RNA molecules, such as small interfering RNAs (siRNAs), antisense oligonucleotides (ASOs), and clustered regularly interspaced short palindromic repeats (CRISPR)-based gene editing, are used. SiRNAs are composed of two strands: the sense strand and the antisense strand. The antisense strand is designed to match the sequence of the target mRNA, enabling it to serve as a guide for the molecule. Once internalized via endocytosis, the siRNA engages with key proteins, including the RNAse III enzyme Dicer, the RNAse Argonaute, and an RNA-binding cofactor, to form the RNA-induced silencing complex loading complex. During this process, the sense strand is eliminated, resulting in the formation of a functional RNA-induced silencing complex (RISC). This mature complex binds specifically to the target mRNA using the complementary antisense strand, thereby silencing gene expression through mechanisms such as target mRNA cleavage and degradation [217]. On the other hand, nucleic acid-based therapeutics can replenish gene expression through the delivery of functional nucleic acids in the form of plasmids or mRNA [218]. While plasmidic DNA expression at therapeutic levels in vivo is still challenging, mRNA-based therapy seems more promising.

#### 6.1.1. MASLD

MicroRNAs (miRNAs) are non-coding single-stranded RNA strands of 20–25 nucleotides that play pivotal roles in diverse physiological and pathological pathways, such as in hepatobiliary cancers, by modulating the expression of downstream genes [219]. Recent research has underscored the pivotal role of these biomolecules in driving MASLD progression to MASH or other complications, owing to their capacity to modulate the expression of genes associated with oxidative stress, lipid metabolism, inflammation, fibrosis, and cell proliferation (extensively described in [220]). Targeting MASH-promoting miRNAs with synthetic molecules offers a promising avenue for therapeutic intervention. In fact, several studies have highlighted an abnormal profile of hepatic and circulating miRNAs in MASH and HCC patients. For instance, in mice treated with a methionine choline-deficient diet (MCD), described in Section 5.1.1, to reproduce features of human MASH, miR-122 was found to be elevated and associated with MASH severity, pointing out this miRNA, which is involved in glucose and cholesterol metabolism, as a potential biomarker for the early detection of hepatotoxic alterations [221]. Importantly, miR-122 knockout mice progressively developed steatohepatitis, fibrosis, and hepatocellular cancer, showing an important role of miR-122 in MASH and HCC development [222]. These mice also presented elevated alkaline phosphatase levels, indicating bile duct injury. In fact, in end-stage PBC patients’ liver tissue, miR-122 was significantly downregulated; on the other hand, this miRNA was found to be upregulated in the serum of PBC patients versus controls, suggesting that context-dependent studies should be performed before any miRNA-based therapeutic strategy is adopted [223,224]. Other miRNAs that have been identified preclinically and clinically as potential therapeutic targets for MASLD and MASH are miR-132-3p, -10b-5p, 22-5p, 103a-3p, and 107 (extensively described in [220]). Studying the underlying mechanisms of miRNA-based regulation is crucial for each liver disease in order to design the appropriate therapy (miRNA mimics or antagomiRs).

ASOs are also being tested in clinical trials for MASLD and MASH [225]. For instance, the safety and tolerability of different doses of AZD2693, a potent patatin-like phospholipase domain-containing 3 (PNPLA3) GalNac-ASO, applied subcutaneously in patients with MASH (NASH), fibrosis Stage 0 to 3, and carriers of the PNPLA3 I48M Risk Alleles, was tested (NCT04483947, clinicalgov.it last accessed on 9 December 2024). PNPLA3 is an important regulator of lipids in hepatocytes and stellate cells. It is involved in the remodeling of phospholipids of lipid droplets in hepatocytes and is regulated by TGF-B to release retinol from retinyl esters during the activation of HSCs, hence implying an important regulatory role of PNPLA3 in NAFLD [226]. The results of the NCT04483947 clinical study were recently presented at the International Liver Congress in Milan (https://www.postersessiononline.eu/173580348_eu/congresos/EASL2024/aula/-LBP_31_EASL2024.pdf, accessed on 10 December 2024). In brief, AZD2693 could efficiently reduce both PNPLA3 I48M mRNA and protein levels in primary human hepatocytes, was well-tolerated in healthy volunteers, and achieved targeted knockdown of PNPLA3 in PNPLA3 I48M homozygous risk allele subjects with subsequent mild reduction in liver fat content, increase in polyunsaturated fatty acids in circulating triglycerides, and decrease in the inflammatory marker C-reactive protein. These data set the basis for a further Phase 2b clinical study (NCT05809934, clinicaltrials.gov, last accessed on 9 December 2024). This study will evaluate the histological improvement of MASH in PNPLA3 risk allele carrier subjects affected by fibrosis without cirrhosis after 1 year. Another clinical study has recently reported the efficacy of a Ligand-Conjugated Antisense (LICA) against diacylglycerol acyltransferase 2 (DGAT2), which regulates triglycerides, in decreasing liver fat, hence leading to improvement in clinical features of MASH (NCT04932512, clinicaltrials.gov, last accessed on 9 December 2024; https://ir.ionis.com/news-releases/news-release-details/ionis-announces-positive-results-phase-2-study-ion224).

Other studies have employed siRNA/GalNAc conjugates to modulate target gene expression in NASH patients. For example, the safety and tolerability of ARO-HSD, a siRNA-based therapeutic that selectively targets HSD17β13 mRNA in the hepatocytes, subcutaneously administered in healthy volunteers and NASH patients, was assessed in a Phase 1 clinical study (NCT04202354, clinicaltrial.gov, last accessed on 9 December 2024). HSD17β13 regulates the metabolism of hormones, fatty acids, and bile acids in hepatocytes, and it has been shown that HSD17β13 loss-of-function results in chronic liver disease protection [227]. HSD17β1 is overexpressed in NAFLD patients’ livers versus controls [228]. The results of the NCT04202354 clinical trial have been published and demonstrate that ARO-HSD was well-tolerated in the study participants, achieving over 75% HSD17β1 mRNA reduction as well as ALT decrease, warranting a Phase II study with a larger cohort of patients [229]. Research for new therapeutic candidates is also underway. One of the latest reports regards the therapeutic silencing of perilipin 2 (PLIN2), a lipid droplet protein inhibiting hepatic lipolysis, in mice fed a high-fat diet, described in Section 5.1.1, to reproduce features of MASLD, and fed a methionine/choline-deficient diet or HFD/high-fructose diet to mimic MASH [230]. Treatment of mice with GalNAc-si*Plin2* led to a reduction in hepatic PLIN2 levels, and a significant improvement in steatosis, liver injury, inflammation, and fibrosis was observed in vivo.

CRISPR-based gene editing also offers promises for reducing steatosis in NAFLD. Preclinical studies reveal that CRISPR/Cas9-mediated knockdown of *SOCS3*, *DUSP1*, and *SIK1* in human adipose-derived mesenchymal stem cells (hADMSC)-derived adipocytes led to a modulation of de novo lipogenesis and palmitate-induced lipogenesis in vitro [231]. Co-culture of hADMSC-derived adipocytes with primary hepatocytes induced a decrease in lipid storage in the latter, suggesting that *SOCS3*, *DUSP1*, and *SIK1* silencing can be beneficial in NAFLD. Human fetal hepatocyte organoids generated in the context of MASLD and MASH, as described in Section 5.2.1, coupled with CRISPR/Cas9 technology, could appropriately model three situations responsible for steatosis (free fatty acid loading, genetic variation (PNPLA3 I148M), and monogenic lipid disorders (familial hypobetalipoproteinemia and abetalipoproteinemia with mutations in *APOB* and *MTTP*, respectively)) and hence, allow drug screening for targeted MASLD therapy [232].

#### 6.1.2. HDV

HDV has a circular, single-stranded RNA genome coupled to a large and small hepatitis D antigen (HDAg), encoded by the open reading frame (ORF) to form the ribonucleoprotein (RNP) packed inside the delta virus genus envelope [233]. HDAg-susceptible host enzymes regulate HDV replication. As HDV can only infect patients with pre-existing HBV infection, an important strategy to target HDV is to block HBV replication or assembly. For instance, nucleic acid polymers (NAP, REP 2139), which block HBV surface antigen (HBsAg) release from infected hepatocytes, hence interfering with HBV subviral particle assembly, have shown promise in clinical studies [234]. REP 2139 is a 40-mer, fully phosphorothioated polyadenosine-cytidine sequence, with incorporation of 5-methylation of all cytosines and 2′O-methyl modification of all riboses. Importantly, combination therapy with REP 2139 and pegylated interferon alfa-2a in a phase 2 trial could control HBV/HDV co-infection, leading to liver functionality restoration, and was well-tolerated and safe when administered in patients [235].

Optimized nuclease-resistant siRNAs are promising as therapeutics to inhibit HBV replication by targeting RNA molecules, such as pregenomic RNA and mRNA, to prevent the formation of covalently closed circular DNA (cccDNA) [236]. Incorporation of phosphorothioate linkages combined with 2′-OH modifications improves siRNA stability, increasing their efficacy at low doses, thus reducing side effects. Another study showed that the addition of 2′-O-guanidinopropyl-modified ribonucleotides enhanced siRNA stability, reduced immune activation, and increased the effectiveness of gene silencing. Delivery of siRNAs to target organs is also an important issue to be addressed. For instance, the attachment of siRNAs to N-acetylgalactosamine (GalNAc) has emerged as a leading approach for liver-targeted therapies. Enhanced GalNAc derivatives exhibit stronger binding to asialoglycoprotein receptors on hepatocytes, allowing for precise liver-specific delivery of siRNAs. Preclinically, co-administration of a melittin-like peptide (MLP) targeting hepatocytes with cholesterol-conjugated siRNA achieved a dramatic reduction in HBV gene expression and viral DNA levels, demonstrating a prolonged therapeutic effect in chronic HBV infection mouse models [237]. Further advancements, such as chemically altered triantennary N-acetylgalactosamine (GalNAc) ligands, facilitated asialoglycoprotein receptor (ASGPR)-mediated delivery of functional siRNAs to liver cells [238]. This research has been translated to the clinical setting. A Phase 2 clinical trial aimed at assessing the safety and efficacy of various doses of ATI-2173 (a liver-targeted phosphoramidate prodrug of clevudine, engineered to enhance anti-HBV activity while minimizing systemic exposure to clevudine) in combination with Tenofovir Disoproxil Fumarate and AB-729 (a potent, selective, subcutaneously administered N-acetylgalactosamine (GalNAc)-conjugated siRNA inhibitor of HBV), or placebo, in individuals with chronic HBV infection and those with HDV co-infection (NCT04847440; source: clinicaltrials.gov; last visited on 5 December 2024). While results from this study have not been posted, it was recently reported that 7 patients with cHBV have discontinued all treatments and showed sustained low levels of HBV DNA and HBsAg for a minimum of 18 months following AB-729 treatment (https://www.natap.org/2023/HBV/050223_03.htm, last visited on 5 December 2024). Thus, the siRNA strategy to suppress HBV to indirectly target and inhibit HDV is very promising.

A CRISPR/cytidine base editor (CBE) system was also evaluated for the ability to eliminate HBV through single-guide RNA (sgRNA) created to edit the codon number 30 of the HBV *S* gene, which encodes HBV surface antigen (HBsAg). This study showed that HBV-positive human hepatoma PLC/PRF/5 cells treated with the treatment of gRNA_S had a 92% reduction in HBsAg secretion and an 84% reduction in intracellular HBsAg without off-target effects [239].

Another significant advancement in the realm of nucleic acid-based therapy for HBV involves the use of ASO treatments. Preclinical research has demonstrated that Bepirovirsen (GSK3228836), a GalNAc-conjugated 2′-O-methoxyethyl gapmer designed to target HBV mRNA and pregenomic RNA, led to a decrease in HBV-derived RNAs, HBV DNA, as well as viral proteins in HepG2A.2.15 cells and in HBV transgenic mice when administered subcutaneously in the latter [240]. Multiple doses of Bepirovirsen injection were safe and led to a sustained response in vivo. Importantly, a Phase 2 clinical trial (NCT02981602, clinicaltrial.gov, last accessed on 6 December 2024) was performed to evaluate the safety and tolerability, as well as the antiviral activity, of multiple doses of injected Bepirovirsen in chronic HBV patients. The results of this study have been published and have demonstrated there was a dose-dependent reduction in HBsAg and HBV DNA following 4 weeks of treatment with Bepirovirsen, with a favorable safety profile, warranting further clinical studies [241]. Researchers have also explored the combined application of siRNAs with ASO-based therapies by combining the siRNA ALG-125903 with the ASO ALG-020579, alongside other anti-HBV agents such as nucleos(t)ide analogs (NAs) and capsid assembly modulators [242]. The results showed that this combo led to a rapid and potent suppression of HBsAg in vivo.

#### 6.1.3. AIH

In the case of AIH, which is caused by multiple factors, the development of ASOs and CRISPR/Cas9-gene modulation is still to come. Therapeutic silencing of genes involved in the pro-inflammatory cascade, such as those encoding for cytokines (e.g., IL-17, IL-6) through siRNAs, may be an approach [243]. For instance, Zhang et al. loaded M2-macrophage-derived exosomes, derived in vitro, with siRNAs targeting receptor-interacting protein kinase 3 (RIPK3), the expression of which was found elevated in the liver and peripheral blood monocytes of AIH patients [244]. They showed that the M2 Exos/siRIPK3 alleviated immune injury by specifically targeting macrophages and suppressing the release of proinflammatory cytokines, such as IFN-g and IL-6, in a mouse model of AIH induced by concanavalin A.

#### 6.1.4. Cholangiopathies (PBC, PSC)

ASOs previously developed in the context of MASH are proving to be useful for PSC. In a preclinical study, it was shown that knockdown of the seven-transmembrane superfamily member three protein (TM7SF3) potentiates HSC activation and exacerbates fibrogenesis by promoting the alternative splicing of the Hippo pathway transcription factor, TEA domain transcription factor 1 (TEAD1), by inhibiting the splicing factor heterogeneous nuclear ribonucleoprotein U (hnRNPU) [245]. Exon 5 skipping in TEAD1 leads to an active form that contributes to the expression of pro-fibrogenic genes, such as *Ctgf* and *Cyr61* [246,247,248]. ASO 56, an ASO designed to inhibit hnRNPU binding on intron 5-exon 5 on TEAD pre-mRNA, was found to decrease the expression of the active TEAD1 in HSCs, causing a reduction in their activation and, consequently, in fibrosis-related gene expression both in vitro and in vivo. Moreover, MASH features were dampened in mice (fed a MASH diet, described in Section 5.1.1) treated with ASO 56. This strategy was also tested in a PSC model with results promising for a future clinical translation (https://liverdiseasenews.com/news/drug-created-reduce-liver-scarring-may-be-psc-treatment-fibrosis/, last visited on 10 December 2024).

SiRNA-based therapeutics are also being developed and tested for PSC treatment. The safety and tolerability of STP707, a nanoparticle containing 2 siRNAs targeting the pro-inflammatory TGF-β1 and COX-2 mRNA, respectively, has been tested in a clinical study on healthy volunteers (NCT05309915, clinicaltrials.gov, and https://sirnaomics.com/en/news-room/press-release/20220404sirnaomics-doses-first-subject-in-phase-i-clinical-trial-of-stp707-for-the-treatment-of-liver-fibrosis-in-primary-sclerosing-cholangitis-2/; last visited on 10 December 2024) with the aim of developing siRNA-based therapeutics for PSC. No results regarding this study have been posted, but STP707 was found to successfully reduce tumor size in patients with different types of solid tumors and at late stages, leading to stable disease, warranting longer-term studies (https://sirnaomics.com/en/news-room/press-release/20230831sirnaomics-announces-successful-phase-i-clinical-study-of-rnai-therapeutic-stp707-for-treatment-of-multiple-solid-tumors/, last visited on 10 December 2024).

Regarding PBC, no siRNA-based therapeutic approaches have been documented. Few studies have reported on miRNAs differentially expressed in PBC versus controls. In this regard, miR-506 was found to be elevated in PBC patients [249]. Mir-506 targets Cl^−^/HCO_3_^−^ anion exchanger 2 (AE2/SLC4A2) and type III inositol 1,4,5-trisphosphate receptor (InsP3R3) in cholangiocytes and can thus develop into a therapeutic target for PBC [250]. However, miR-506 has been shown to act as a tumor suppressor, pointing to the need for caution while developing anti-miR-506 therapeutics for PBC [251,252]. A two-phase strategy can be developed, as reported by Yang et al., that is, the targeting of immune-mediated pathogenesis and inflammation in the early phases and fibrogenesis in the late phases [253]. Targeting pathways associated with bile acid homeostasis (e.g., FXR, TGR5) using ASOs and gene editing can also result in efficient therapeutic strategies.

Data on safety, efficacy, and patient outcomes are not yet available for most of the above-mentioned Phase 2 clinical trials. However, several Phase 2 and 3 clinical trials have employed siRNAs and ASOs as therapeutic tools for CHDs and have provided encouraging results [254,255]. Regarding FDA-approved siRNA drugs for CHDs, the hydroxyacid oxidase 1-targeting and GalNAc-conjugated siRNA, lumasiran, for instance, were well tolerated in Phase I/II, Phase II and Phase III studies in subjects affected by primary hyperoxaluria type 1 [256]. Treatment with lumasiran led to an improvement in clinical parameters, including urinary oxalate levels, in patients. The ASO Bepirovirsen targeting all HBV mRNAs also showed a good safety profile in chronic hepatitis B patients and a dose-dependent reduction from baseline in HBsAg and HBV DNA after 4 weeks of treatment [241].

### 6.2. Other Potential Molecular Therapies for CHD

Stem cell therapy and EV-based therapies are also promising for CHD treatment. Stem cell-based therapies aim to regenerate damaged liver tissues and restore function. Mesenchymal stromal/stem cells (MSCs) are the most studied cell types in CHD due to their ability to home efficiently to injury and inflamed tissues and to the documented benefit obtained by inflammation modulation, tissue repair, and anti-fibrotic action [257,258]. Conversely, MSCs can exert immune-conferring functions in diseases where the immune system is compromised, such as due to chronic viral infections. MSCs can act as carriers of therapeutic drugs capable of modulating the immune response of these patients. In AIH, where excessive immune responses lead to the attack of the hepatocytes, MSCs may act in a paracrine way, for instance, by releasing immunomodulatory molecules such as FasL, iNOS, and TGF –β, to induce self-tolerance by inhibiting lymphocyte activity and promoting Treg formation [259]. Several clinical studies have tested the safety, tolerability, and therapeutic effect of MSCs for AIH and other CHDs, as listed in the present review (Table 2).

MSC-based therapy can also improve MASLD features, such as lipid and glucose metabolism, inflammation, and fibrosis, thus impeding the progression to MASH in preclinical models (extensively described in [259]). A recent study also shows that human umbilical cord-derived MSCs can attenuate diet-induced obesity and MASH-related fibrosis in mice [260]. Treatment with MSCs significantly decreased the body weight of mice fed a Western diet compared to controls. Glucose tolerance and insulin sensitivity were markedly improved, and liver steatosis, inflammation, and fibrosis were significantly reduced by intravenously administered MSCs, which lowered the expression of genes involved in fatty acid metabolism (Acot1) and the expression of *Cyp4a10* and *Cyp4a14* involved in the PPAR signaling pathway. MSCs can also exert therapeutic effects in MASLD and MASH models by modulating the polarization and activity of liver macrophages [261]. Clinical studies are yet to be undertaken to investigate the therapeutic potential in MASLD and MASH patients. In certain cholangiopathies, MSCs are already being tested in patients following encouraging preclinical results. For instance, a PSC patient received a bone marrow-derived MSC infusion through the hepatic artery, which led to an improvement in bilirubin levels at 6 months following treatment [262]. Regarding PBC, two clinical studies have been performed hitherto (Table 2) with MSCs. No obvious side effects were observed, indicating a good safety and tolerability of the injected cells [262]. In one of the studies enrolling UCDA-resistant PBC patients, the injection of bone marrow-derived MSCs led to an improvement in serum ALT, AST, GGT, and IgM levels; CD8+ cell numbers decreased while Tregs increased in the peripheral blood, confirming the immunomodulatory and therapeutic functions of MSCs [263]. More long-term studies are needed to show the therapeutic potential of these cells in the treatment of this disease.

EVs are heterogeneous, lipid-bilayered structures found in all body fluids that transport biomolecules, such as lipids, metabolites, nucleic acids (such as miRNA, mRNA, and DNA), and proteins (including cell surface receptors, signaling molecules, transcription factors, enzymes, and RNA-binding proteins), to target cells. EVs exist in different subtypes, classified according to their synthesis and release mechanisms, and include microvesicles, exosomes, and apoptotic bodies, as well as large oncosomes, migrasomes, ectosomes, exomeres, supermeres, and membrane particles [264]. EVs are also a source of biomarkers for CHDs [3].

**Figure 2:**
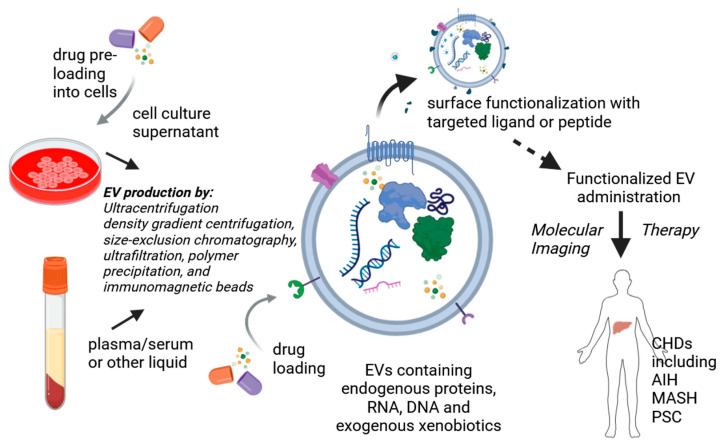
Functionalization of EVs for the treatment of CHDs. Exogenous xenobiotic loading can be achieved either by treating cells with the drug of interest, resulting in EVs produced by the cultured cells being naturally loaded with the compound, or through direct EV loading methods, such as electroporation or incubation. Functionalized EVs can be used as theranostic agents in patients with CHDs.

In liver disease therapy studies, MSC-EVs are primarily obtained from sources such as umbilical cords, bone marrow, and adipose tissue. EVs derived from MSCs, for instance, harbor therapeutic molecules that can slow down the progression of liver diseases. Importantly, MSC-EVs derived from different sources harbor different biomolecular contents and can be employed in specific contexts. For example, proteins related to injury regulation, such as DKK-1, GRO-α, IL-8, and IGFBP-3, were prominently elevated in EVs derived from adipose tissue. In contrast, EVs sourced from bone marrow exhibited heightened expression of proteins associated with bone formation and new blood vessel growth, including ANG-2, BDNF, IFN-γ, IL-1α, KLK-3, and RETN [265]. Of great interest is the fact that EVs show high liver tropism during biodistribution studies in vivo, thus facilitating their use for CHD therapy. A recent study using Positron Emission Tomography (PET) showed how deferoxamine-conjugated and Zr-89 radiolabeled EV clearance primarily occurs in the liver after a maximum permanence of 72 h in the circulation [266]. There was a differential hepatic uptake of labeled EVs obtained from different sources; EVs derived from human macrophages showed a higher uptake (approximately 4 times more) in the liver with respect to MSC- or melanoma cell line-derived EVs, probably due to the type of proteins located on the surface of these EVs. These results show how it is crucial to choose the source of EVs, as well as the type of functionalization, not only to maximize the hepatic uptake of EVs but also for imaging in vivo. EV-based theranostics (for therapy and diagnosis) can be employed in the case of CHDs (Figure 2) [267]. In the case of AIH, for example, bone marrow-derived MSC-EVs, non-modified versus miR223-enriched or -depleted, were tested in the experimental AIH mouse model generated by hepatic S100 injection [268]. It was found that both non-modified EVs and those enriched with miR223 attenuated liver injury in these mice through the reduction in the NLRP3-Caspase 1 signaling pathway. These MSC-EVs, engineered to contain high levels of the immunomodulatory miRNA, miR-223, could lower the serum levels of IL-1β, IL-6, and IL-17, and increase those of IL-10, correlated to a decrease in Th17 cells and an increase in Tregs [269]. In a mouse model of NASH (MCD-diet treated), intravenous injection of human umbilical cord-derived MSC-EVs (exosomes) resulted in weight loss and reduction in liver damage, as well as a decrease in TNF-α, IL-6, and IL-1β pro-inflammatory cytokine levels in the plasma [270]. These EVs could also restore the lipid metabolism regulator, PPAR-α, activity, which is reduced upon treatment with the MCD diet in mice. The *Mdr2*^−/−^ mice present features of human PSC. Bone marrow-derived MSC-EVs intravenously administered in multiple doses for 3 weeks showed anti-fibrotic and anti-inflammatory effects in these mice, thus halting PSC progression [271]. A reduction in serum ALT, bile acids, and ALP, as well as in hepatic CD8+ T cells and granulocytes, was observed. MSC-EVs thus reduce metabolic disorders, dampening inflammation, blocking the progression of fibrosis, and enhancing liver regeneration, indicating that the EV system can be further harnessed for the treatment of AIH, NASH, as well as other CHDs (Figure 2).

Although natural MSC-EV offers numerous potential benefits, several intrinsic challenges hinder its clinical application. Issues such as variability, limited production, and swift clearance in vivo can impact both its quality and therapeutic effectiveness. To address these concerns, researchers are developing engineered MSC-EVs to enhance their therapeutic performance and persistence within the body [272]. MSC-EV exhibits remarkable adaptability and can be significantly influenced by external factors. Consequently, diverse in vitro preconditioning strategies are being investigated to boost their therapeutic capabilities and optimize their in vivo behavior. These approaches include altering MSC culture environments, introducing exogenous cytokines or drugs, modifying EV cargo and membrane proteins, and refining delivery systems and administration routes [272]. Hypoxia, for instance, can increase mouse bone marrow-derived MSCs’ capacity to release EVs containing miR-182-5p with respect to conventionally cultured MSCs [273]. MiR182-5p increased the regenerative capacity of the liver after partial hepatectomy in mice by targeting forkhead box transcription factor 1 (FOXO1) in macrophages, hence dampening the FOXO1/toll-like receptor 4 (TLR4) signaling pathway and promoting an anti-inflammatory response. Culturing MSCs in 3D to enhance EV production and anti-fibrotic potential and enhancing the half-life of EVs are some of the other strategies to increase the EVs’ therapeutic potential and permanence in vivo. In regard to the latter approach, enhancing liver targeting and immune evasion by altering the surface molecules of MSC-EV, encapsulating MSC-EV within biomaterials to extend their half-life, and optimizing delivery methods to minimize the distribution of MSC-EV to non-target tissues in vivo have been reported [272].

Apart from MSCs, human liver stem cells (HLSCs) are also a good source of EVs for the treatment of liver diseases. For instance, Bruno et al. showed that HLSC-derived EVs could alleviate liver fibrosis and inflammation, leading to improved liver function in a mouse model of NASH [274]. The beneficial effect was attributed to the presence of cytokines, growth factors, and miRNAs, like miRNA-29a, the let-7 family, miRNA-30a, miRNA-24, and miRNA-21, that are known to modulate the expression of the fibrogenesis-related Collagen I, Snail, and the FAS ligand genes. More research is needed regarding the best EV source, the EV cargo definition, as well as strategies to specifically target the diseased hepatic cell type in order to successfully resolve human CHDs.

## 7. Conclusions

Preclinical studies have been tremendously helpful in designing new, safe, tolerable, and personalized therapies for human CLDs before they progress to the cirrhotic stage. The translation of newly identified molecular therapies into clinical practice offers a great breakthrough for CLDs, but some aspects need further research. Nanotechnology, not discussed in this review, corroborates this important progress in modern therapeutics for complex liver pathologies. Future efforts will have to address the problems of optimal delivery systems, toxicity reduction, and ensuring broad accessibility in longer-term studies. Nanoparticle-based systems can be implemented not only for drug delivery to liver cells in a highly efficient and specific way but also for imaging for diagnostic purposes when coupled with probes (theranostic nanosystems). A diverse range of substances, such as small chemical compounds, proteins, peptides, nucleic acids, and RNA oligonucleotides, can be incorporated into these nanoparticles to enable both specific and non-targeted delivery methods. Some of the nanoparticles employed for CHD treatment and diagnosis are liposomes, polymeric nanoparticles, and micelles [275]. Superparamagnetic IO nanoparticles (SPIONs) with α-tocopheryl-polyethyleneglycol-succinate (TPGS) surface modification were found to be safer with respect to the non-modified version in terms of cytotoxicity, but the accumulation of the SPION-TPGS in the liver resulted in genotoxicity, which was reversed upon treatment suspension [276]. SPIONs, such as the strawberry-like Fe3O4-Au nanoparticles, were employed as dual-modality contrast agents for CT and MRI imaging in in vivo studies in rats with fatty liver (generated using the Lieber-DeCarli formulation of liquid diets and by gradually introducing ethanol), cirrhosis (induced by CCl_4_ injection), and HCC (obtained by subcutaneously injecting Walker-256 cells) [277]. These nanoparticles demonstrated excellent biocompatibility and could distinguish among disease stages, making them promising candidates for future clinical applications. There are, however, concerns regarding possible off-target effects, delivery barriers, cytotoxicity or genotoxicity, scaling up, and the cost-effectiveness of the CHD therapeutic drugs, which need to be accurately addressed in preclinical studies and clinical trials. The concept of developing vaccines for chronic liver diseases represents a frontier in biomedical research, focusing on both infectious and non-infectious etiologies. While vaccines for viral hepatitis, such as HBV and HAV, have already demonstrated the transformative impact of immune prevention, the unmet need for HCV and HDV vaccines highlights ongoing challenges in combating viral liver diseases. Beyond infectious causes, there is growing interest in leveraging vaccine technology to target CHDs such as autoimmune liver diseases (AILDs) and metabolic liver diseases.

## Figures and Tables

**Table 1:** Classifications of chronic liver diseases considered in the present review: characteristics, traditional therapy, emerging, and future targeted treatments.

Disease	Etiology	Characteristics	Traditional Treatment	Emerging Treatment	Future Targeted Treatment
MASLD	Dysmetabolic	Presence of liver steatosis (≥5%) +T2DM, obesity or other metabolic abnormalities	Lifestyle, GLP1-RA, Resmetirom	Emirican, Selonsertib, Vitamin D, Setanaxib, probiotics, PRI-724, OCA, Tropifexor, Aldafermin, Simtuzumab	miR-122, miR-132-3p, miR-10b-5p, miR-22-5p, miR-103a-3p, miR-107, AZD2693, ARO-HSD, SOCS3, DUSP1, SIK1
HDV	Viral	Infection is possible only in HBV+ patients	PEG-IFNα, Bulevirtide	PRI-724	REP 2139, ATI-2173, AB-729, Bepirovirsen
AIH	Autoimmune	Frequently associated with other immune-related disease	Prednisone and AZA	-	M2 Exos/siRIPK3
PSC	Unknown	Progressive inflammation and fibrosis of biliary ducts	UDCA	Probiotics, ICG-001, PRI-724, OCA, Tropifexor, Simtuzumab	ASO 56, STP707
PBC	Autoimmune	Immuno-mediated destruction of biliary ducts	UDCA, OCA	Setanaxib, ICG-001, PRI-724, Tropifexor	ASO 56, miR-506

MASLD: metabolic dysfunction-associated steatotic liver disease; T2DM: type 2 diabetes mellitus; GLP1-RA: glucagon-like peptide-1 receptor agonists; HDV: hepatitis D virus; HBV: hepatitis B virus; AIH: autoimmune hepatitis; AZA: azathioprine; PSC: primary sclerosing cholangitis; UDCA: ursodeoxycholic acid; PBC: primary biliary cholangitis; OCA: obeticholic acid.

**Table 2:** Examples of clinical trials with stem cells for the treatment of chronic liver diseases are described in the present review.

MSC Source	Disease	Type of Intervention/Results	Dose/Duration/Administration Route	Aim/Expected Outcome	Study Number/Country
Umbilical cord	AIH and PBC	Interventional/Phase 1/2/results N/A	1 million cells/kg body weight for 12 weeks/I.V. once per 4 week	Evaluation of efficacy and safety/Liver Histology change	NCT01661842/NCT01662973 China
Bone marrow/allogeneic	PBC	Interventional/Phase 1/results N/A	5–50 million cells /kg body weight/24 months if first dose functional/I.V.	Evaluate efficacy and safety of MSCs/improvement in symptoms, survival and side effects	NCT01440309/China
Umbilical cord	AIH and PSC	Adaptive, single arm, multi-centre, phase IIa/active	0.5, 1.0, 2.5 million cells/kg body weight/I.V. single dose	Reduce inflammation/Dose safety and treatment activity determination	NCT02997878/UK
Umbilical cord	PSC	Interventional/Phase 1/2/Results N/A	N/A/Infusion at day 0, 7, 14, and 21/Intra-arterial	Evaluate adverse effects/changes in biliary lesions and inflammation	NCT03516006/China

Data have been taken from clinicaltrials.gov (last accessed on 21 November 2024). N/A: not applicable.

## Data Availability

No new data were created.
